# Commentary: Nursing education as intervention—reducing caregiver burden and enhancing outcomes

**DOI:** 10.1177/17449871251384950

**Published:** 2025-10-30

**Authors:** Sarah Bekaert

**Affiliations:** Associate Professor, Oxford School of Nursing and Midwifery, Oxford Brookes University, Oxford, UK

**Commentary to:** The effect of planned education given to mothers of children with hydrocephalus on care burden and anxiety: a randomised controlled study (Keklik et al., 2026).

This randomised controlled trial by Keklik et al. (2026) addresses an important gap in paediatric neurosurgical care: the role of structured health education for mothers of children with hydrocephalus by nurses. The authors set out how hydrocephalus, the most common indication for paediatric neurosurgery, is primarily managed through shunt placement. Yet, complications, particularly infections occurring within the first 6 months, remain common, costly, and distressing for families. They describe how beyond medical risks, mothers face substantial physical, emotional, and financial challenges; increasing caregiving burden and anxiety. Although prior evidence suggests education can improve condition management, few studies have examined its combined effect on caregiver well-being and shunt-related outcomes.

This study was conducted in two Turkish university hospitals, randomising 40 mothers of infants (0–1 years) undergoing ventriculoperitoneal shunt surgery into intervention and control groups. The intervention group received structured, manual-based education plus weekly follow-up over 4 weeks, whereas controls received routine care. Outcomes explored included caregiver burden, anxiety, and shunt-related complications.

Findings showed mothers in both groups experienced some caregiving burden and moderate anxiety, with no significant differences in scale scores. However, positive correlations were observed between burden and anxiety, particularly in the control group. More striking were differences in shunt-related outcomes: by 1 month, only 15% of children in the intervention group experienced shunt infections compared to 50% in controls (*p* = 0.018). Mothers receiving education also reported fewer problems related to their child’s crying, nausea, swelling, head positioning, and trauma prevention. These results underscore the protective role of structured education in reducing preventable complications and enhancing caregivers’ ability to meet their child’s needs.

Illustrating Dorothea Orem’s Self-Care Deficit Nursing Theory (Orem et al., 2001), this study highlights the nurse’s role in supporting mothers in meeting their child’s care needs. This is a key role where nurses are increasingly seeking to evidence impact, for example studies by Yip et al. (2021) and Dawood et al. (2025) in this journal. The commentary by Bekaert (2025a) on Dawood et al.’s (2025) study emphasises that health education is a core nursing role. In Keklik et al.’s study here, the structured education provided to mothers aligns with this theoretical perspective by enhancing their ability to perform care for their child, recognise early signs of complications, and make informed caregiving decisions.

Actively involving families in care decisions also aligns with Anne Casey’s Family-Centred Care Model (Casey, 1993). Butler and McCreaddie (2015) provided a review of family-centred care research in paediatrics, highlighting the significance of involving families in care decisions and recognising them as experts in their child’s needs. This supports the emphasis in this study on family-centred approaches in paediatric neurosurgical care. Casey’s model emphasises that the family is central to a child’s healthcare. It encourages collaboration, respect and empowerment, with families actively participating in care decisions and management. By recognising parents as experts in their child’s needs, family-centred care guides nurses to educate, support and involve families, improving both child and caregiver outcomes.

The study has several strengths, including its randomised design, structured intervention and focus on an often-overlooked caregiver group. Clinically, the findings highlight the vital role of nurses in educating and supporting families. Providing structured education empowers mothers to recognise complications early, manage daily care and develop care competencies for their child. Overall, this trial provides compelling evidence that maternal education is a low-cost, high-impact intervention in paediatric hydrocephalus care. It reinforces the nurse’s dual role in providing direct care while also facilitating family provided care, ultimately improving child outcomes, reducing preventable complications, and supporting caregiver well-being. Drawing on nursing theory to articulate this vital role enables nurses to articulate their professional contributions and apply theoretical frameworks to guide interventions to support caregivers effectively (Bekaert, 2025b).
